# Whole-Genome Comparative Analysis Reveals Association Between *Salmonella* Genomic Variation and Egg Production Systems

**DOI:** 10.3389/fvets.2021.666767

**Published:** 2021-07-12

**Authors:** Hamid Reza Sodagari, Shafi Sahibzada, Ian Robertson, Ihab Habib, Penghao Wang

**Affiliations:** ^1^School of Veterinary Medicine, College of Science, Health, Engineering and Education, Murdoch University, Murdoch, WA, Australia; ^2^Department of Veterinary Medicine, College of Food and Agriculture, United Arab Emirates University, Al Ain, United Arab Emirates; ^3^Medical, Molecular and Forensic Sciences, College of Science, Health, Engineering and Education, Murdoch University, Murdoch, WA, Australia

**Keywords:** *Salmonella*, genomics, egg production system, antimicrobial resistance, virulence, genome environment interaction

## Abstract

Non-typhoidal *Salmonella*, particularly *Salmonella enterica* serovar Typhimurium (*S*. Typhimurium), is the predominant endemic serovar in the Australian egg production industry and is one of the most frequently reported serovars in foodborne infections in Australia. This study was conducted to investigate the genomic characteristics of *Salmonella* isolated from retail table eggs in Western Australia and to identify the impact of production systems on genomic characteristics of *Salmonella* such as virulence and antimicrobial resistance. A total of 40 non-typhoidal *Salmonella* isolates [*S*. Typhimurium isolates (*n* = 28) and *Salmonella* Infantis isolates (*n* = 12)] sourced from retail eggs produced by different production systems (barn-laid, cage, and free-range) in Western Australia were sequenced by whole-genome sequencing. The isolates were *de novo* assembled, annotated, and analyzed. The results indicated an association between *Salmonella* genomic variation and the system used to raise poultry for egg production (*p*-value < 0.05). All but one of the *S*. Infantis isolates were recovered from eggs collected from poultry raised under barn and cage production systems. A higher proportion (83.3%) of *S*. Typhimurium isolates were recovered from the eggs produced by free-range production system as compared with those produced under barn (76.9%) and cage production systems (53.3%). Our analysis indicated that *Salmonella* isolated from the eggs produced by barn and cage production systems had more virulence genes than the isolates of the free-range produced eggs. A low carriage of antimicrobial-resistant gene was detected in the isolates of this study. We have built a *Salmonella* genomics database and characteristics-linked gene pools to facilitate future study, characterization, and tracing of *Salmonella* outbreaks.

## Background

*Salmonella* are gram-negative bacteria known for more than 100 years to cause foodborne illness in humans. *Salmonella* are considered host-adapted to many wild and domesticated animals, based on their international distribution and high prevalence in poultry, pigs, and sheep (1). Among 2,600 identified *Salmonella* serovars (2), non-typhoidal serovars have been recognized as the source of 550 million foodborne illnesses annually in the world with 230,000 deaths every year in the world (3); however, many of these *Salmonella*-associated illnesses are preventable with appropriate interventions. Foodborne disease surveillance can be used to gather evidence to help identify emerging strains and resistance that could help in designing appropriate control measures and to evaluate the efficacy of interventional efforts.

In Australia, an estimated 5.4 million cases of foodborne disease occur annually, costing an estimated $1.2 billion per year (4). A number of different *Salmonella* serovars have been isolated in humans and food animals in Australia; however, *Salmonella enterica* serovar Typhimurium (*S*. Typhimurium) is the predominant endemic serovar in the Australian egg production industry and is the most frequently reported serovar in foodborne infections (5, 6). Between 2011 and 2014, 128 outbreaks of *S*. Typhimurium in humans resulted in 2,343 cases with 347 requiring hospitalizations due to consumption of raw eggs and raw egg-related products in various food preparation settings across Australian states/territories, particularly in New South Wales, Queensland, and Victoria (7). Recently, a significant preference of Australian consumers for eggs from cage-free production systems (free-range and barn-laid) has been observed, due to the perception that non-cage production systems produce safer and higher-quality eggs (8) and to public concerns for animal welfare (9).

Over the past decade, Western Australia (WA) has experienced a higher rate of occurrence of human *Salmonella* infection, for both *S*. Typhimurium and non-Typhimurium infections, when compared with the overall national average (10). Better strategies for preventing and managing *Salmonella* outbreaks are of critical importance, and these require improved understanding of the genetic characteristics linked to the mutation, serovar, virulence, and antimicrobial resistance of *Salmonella*. Genomic and metagenomic researches on bacteria play an important role in public health and food safety. The increasing use of whole-genome sequencing (WGS) of *Salmonella* isolates from outbreaks provides a rapid, highly accurate, and discriminatory source tracing and identification of strains. However, there are limited surveillance data and resources available in WA, which have resulted in a lack of detailed understanding of the *Salmonella* outbreaks that occurred in WA.

There have been studies in the USA on the safety of non-cage egg production systems, and it has been hypothesized that non-cage production systems may lead to higher likelihood of exposure to pathogens by chickens, including *Salmonella* (11, 12), due to higher exposure of layer flocks to the outdoor environment, pests, and wildlife vectors. The recent expansion of non-cage production systems has raised concerns about the potential increase in pathogens,such as *Salmonella*, on eggs arising from contaminated farm environments (13). We isolated *Salmonella* strains from eggs available through retail supermarkets in metropolitan Perth, the capital of WA, as previously described (14). Apart from confirming serovar diversity and multilocus sequence types (MLSTs), we conducted WGS to form a better understanding of the genomic mechanisms of the *Salmonella* isolates. In total, we sequenced 40 isolates consisting of three different egg production systems (barn-laid, caged, and free-range). The goals of the study include (1) understanding the genomic characteristics (serovar and MLST) of the *Salmonella* from the WA egg industry and (2) assessing the association between production systems and the genomic characteristics of *Salmonella* such as mutation, virulence, and antimicrobial resistance. This study also indicated the likelihood impact of the environment in driving variations in *Salmonella* genomes and thus forms a basis for devising more effective guidelines and recommendations for industry to better manage the risks of public health in *Salmonella* outbreaks.

## Materials and Methods

### Sampling

We isolated *Salmonella* strains from egg samples (each containing one dozen eggs, totalling 2,400 eggs of 200 dozen packages) purchased from different supermarket chains across Perth residential suburbs. The sample size was calculated as 101 using previously described pooled sampling with Epitools online for uncertain test sensitivity and specificity (15). The estimated true prevalence was considered at precision level of 5% and desired confidence level of 95%, assuming 90% sensitivity and specificity of the test. Subsequently, we collected 200 pool samples to further minimize bias and to represent diversity of retail chain. Details of sample collection and *Salmonella* isolation were described in our previous investigation (14). The proportion of samples from each production system was targeted at ~50% free-range (93 dozen packages), 30% cage-laid (68 dozen packages), and 20% barn-laid (39 dozen packages). These sampling ratios of the production systems were chosen in an effort to reflect the recent egg production demand proportion in Australia (16).

### Isolation, Identification, and Serotyping of *Salmonella*

*Salmonella* isolation was performed according to the ISO 6579-1:2017 standard and followed the procedure described by (17). In summary, the bag containing crushed shells was weighed and mixed a corresponding volume of Buffered Peptone Water (BPW) (Oxoid, Hampshire, UK) to obtain sample-to-diluent ratio at 1:9. Then the mixture was homogenized in a stomacher for 1 min. The bag containing the pooled egg contents of the same sample unit was first blended in a stomacher for 2 min in 25 ml and was homogenized for 1 min with 225 ml of BPW. Both of the homogenized crushed shells and contents were incubated at 37°C for 48 h. After pre-enrichment of the incubated homogenate, 1 ml was inoculated into Muller-Kauffman Tetrathionate Novobiocin Broth (MKTTn) (Oxoid, Hampshire, UK), and also from the same homogenate 0.1 ml was spotted (three drops) on the surface of Modified Semi-solid Rappaport Vassiliadis (MSRV) (Oxoid, Hampshire, UK). MKTTn was incubated for 24 h at 37°C, while MSRV was incubated at 41.5°C and checked after 24 h for a migration zone (turbid, white halo, with radius larger than 10 mm). MSRV plates with no migration zone after 24 h were checked again after 48 h. Streaks from both MKTTn broth and MSRV media were applied on Xylose Lysine Deoxycholate (XLD) agar (Oxoid, Hampshire, UK) and Brilliant Green (BGA) agar (Oxoid, Hampshire, UK), which were then incubated at 37°C for 24 h. Presumptive (up to five) colonies with suspected *Salmonella* morphology were selected from both selective media and transferred into Nutrient Agar (Oxoid, Hampshire, UK) plates. After incubating Nutrient Agar plates at 37°C for 24 h, well-isolated colonies were confirmed to species level using matrix-assisted laser desorption ionization–time-of-flight mass spectrometry (MALDI-TOF MS) using the Microflex instrument (Bruker Diagnostics, Berlin Germany). All confirmed *Salmonella* isolates (up to five isolates per positive sample) were sent for serotyping (Kauffmann-White-Le Minor scheme) by a nationally accredited reference laboratory (PathWest Laboratory, Perth, WA, Australia). Isolates from confirmed positive egg samples were stored at −80°C till further use. A total 40 non-typhoidal *Salmonella* isolates were selected for WGS based on diversity in production systems.

### Whole-Genome Sequencing

DNA was extracted using the BIOLINE DNA extraction kit (ISOLATE II, Genomic DNA Kit) according to the manufacturer's instructions. Library preparation was performed using an Illumina NexTera® XT library preparation kit (Illumina, San Diego, CA, USA) as per manufacturer's instructions. The library preparations were sequenced on an Illumina NextSeq platform using a mid-output 2 × 150 kit.

### Genome Assembly

All the raw sequencing reads were analyzed by using FastQC (version 0.10.1) and MultiQC ver 1.8 (18) to check for read quality. To alleviate contamination from adaptor and vector sequences, the raw read sequences were examined against a comprehensive Illumina adaptor sequence and contaminant library created in-house; and adaptor sequences were removed from the reads when detected. If a read had more than 30 base pair (bp) contaminant sequences, reads were considered problematic and discarded. The raw reads were further processed to remove PCR artifact: only one copy was retained for exactly stacked reads.

The filtered reads were *de novo* assembled using SPAdes software ver 3.11.1 (19) in paired-end mode. Default parameters for SPAdes were used to generate contigs; error correction and Kmer sizes were set to auto. The contigs files were scaffolded by using SSPACE ver 3.0 (20), minimum scaffold size was set to 300 bp, and expected insert size was set to 150 bp with minimum allowed error of 50%.

All read data generated in this study have been deposited in the National Center for Biotechnology Information (NCBI) Sequence Read Archive, and the 40 whole-genome-sequenced *Salmonella* isolate accession numbers are in a continuous serial between SAMN12097892 and SAMN12097931 (project accession number PRJNA549805).

### Serotype, Multilocus Sequence Type, and Virulence Gene Identification

The assembly genomes were uploaded to the Centre for Genomic Epidemiology (http://www.genomicepidemiology.org/) to screen for serotype using SeqSero 1.2 (21), MLST by using MLST ver 1.8 (22), and the contents of virulence genes by VirulenceFinder ver 2.0 (23). The antibiotic resistance genes were also identified by using ResFinder ver 3.1 (23) and NCBI's curated Bacterial Antimicrobial Resistance Reference Gene Database (NCBI, https://www.ncbi.nlm.nih.gov/bioproject/PRJNA313047).

### Genome Annotation and Comparative Analysis

Prokka ver 1.1.2 (24) was used to annotate the gene and microRNA contents of the genome assemblies with bacteria kingdom as annotation model. Standard annotation files, including GFF and GBK annotation files, were generated by running Prokka. *Salmonella* pathogenicity islands (SPIs) were detected with SPIFinder ver 1.0 with default parameters (25) and by BLAST against known SPIs. Prophage regions were identified with PHASTER ver 1.0 (26). Pseudogenes were determined with Pseudofinder ver 0.10 (27) with standard parameters and length 0.8. Genomic rearrangements were detected with progressive Mauve ver 2.4.0, with standard parameters (28). Roary pipeline ver 3.11.2 (29) was used to perform whole-genome comparison and generate a pan-genome of the sequenced *Salmonella* isolates at default parameter set. Scoary ver 1.6.16 (30) and R ver 3.3.2 (9) were used to evaluate the significance of association of the genes identified in the pan-genome with different factors, including serovar and production systems.

### Phylogenetic Analysis

From the phylogenetic analysis, single copy gene families, and multigene families that are conserved among species are identified. Lineage-specific genes, which may contribute to species-specific phenotypes, are determined. Phylogenetic tree of *Salmonella* isolates included in the pan-genome analysis was inferred using maximum likelihood approach (PhyML ver 3.3 under smart model selection) (31) on the single copy orthologous genes identified by Roary with 100 bootstrap replicates. The phylogenetic trees were visualized by using R package “ggtree” ver 2.2.4 (32). The phylogeny was inferred as unrooted and without using outgroups.

### Pathway

Pathway analysis was performed by using the Kyoto Encyclopedia of Genes and Genomes (KEGG) database (33). Annotated genes were supplied to KEGG database through the online interface to map to functional pathways. Identified virulence genes and antimicrobial-resistant genes were searched to identify the pathways they are likely to be involved with by using KEGG database. The associated pathways were visualized by KEGG online access interface.

### Statistical Analysis

The basic genome assemblies' statistics were calculated in R using custom R scripts. The association analysis between *Salmonella* pan-genes and other egg production factors including production system and serotype were performed using Scoary (https://github.com/AdmiralenOla/Scoary). Scoary employs several procedures to filter and perform the association (30). Bonferroni-adjusted *p*-values ≤ 0.05 were used as significance cutoff for Scoary-based association results. Fold change was also used as the secondary criterion in determining the significance of the association. All the downstream statistical analysis and visualization were performed in R using custom R scripts. R packages “ggplots” (34) were used for exploratory analysis; package “ggtree” was used for plotting the phylogenetic tree; “pathfindR” (35) was used for pathway analysis.

## Results

### Genome Assembly and Annotation

All 40 *Salmonella* isolates were assembled to a near-complete form with an average genome size of 4,825,017 bp. The genomes were assembled at high continuity with an average N50 of 257,277 bp, the largest N50 achieved for a single isolate is around 460,000 bp, and the average number of scaffolds is 76. The complete list of the basic statistics of the assemblies of all isolates is given in Supplementary Table 1. The detailed statistics of the assembly of one sample S30, a randomly selected retail isolate from an egg produced in a caged production system, is given in Supplementary Figure 1 as an example to demonstrate the quality of the assembly.

**Figure 1:**
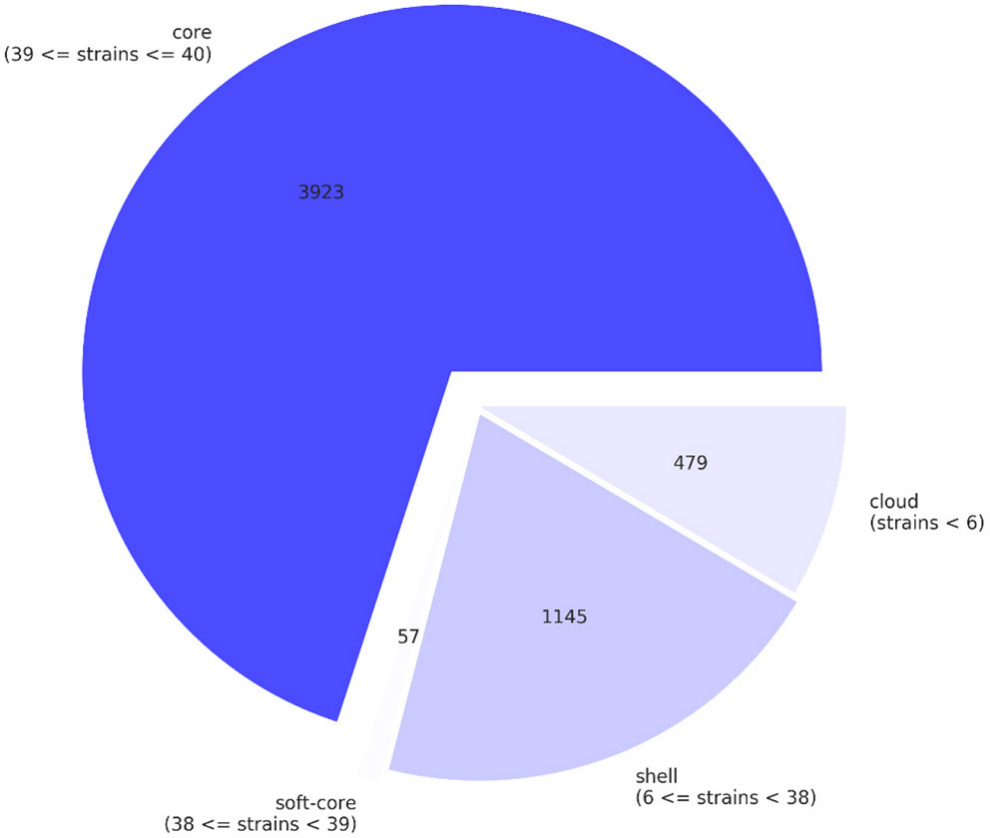
The pan-genome of the retail *Salmonella* isolates, core genes, shell genes, and cloud genes were identified by comparing the isolates.

**Figure 2:**
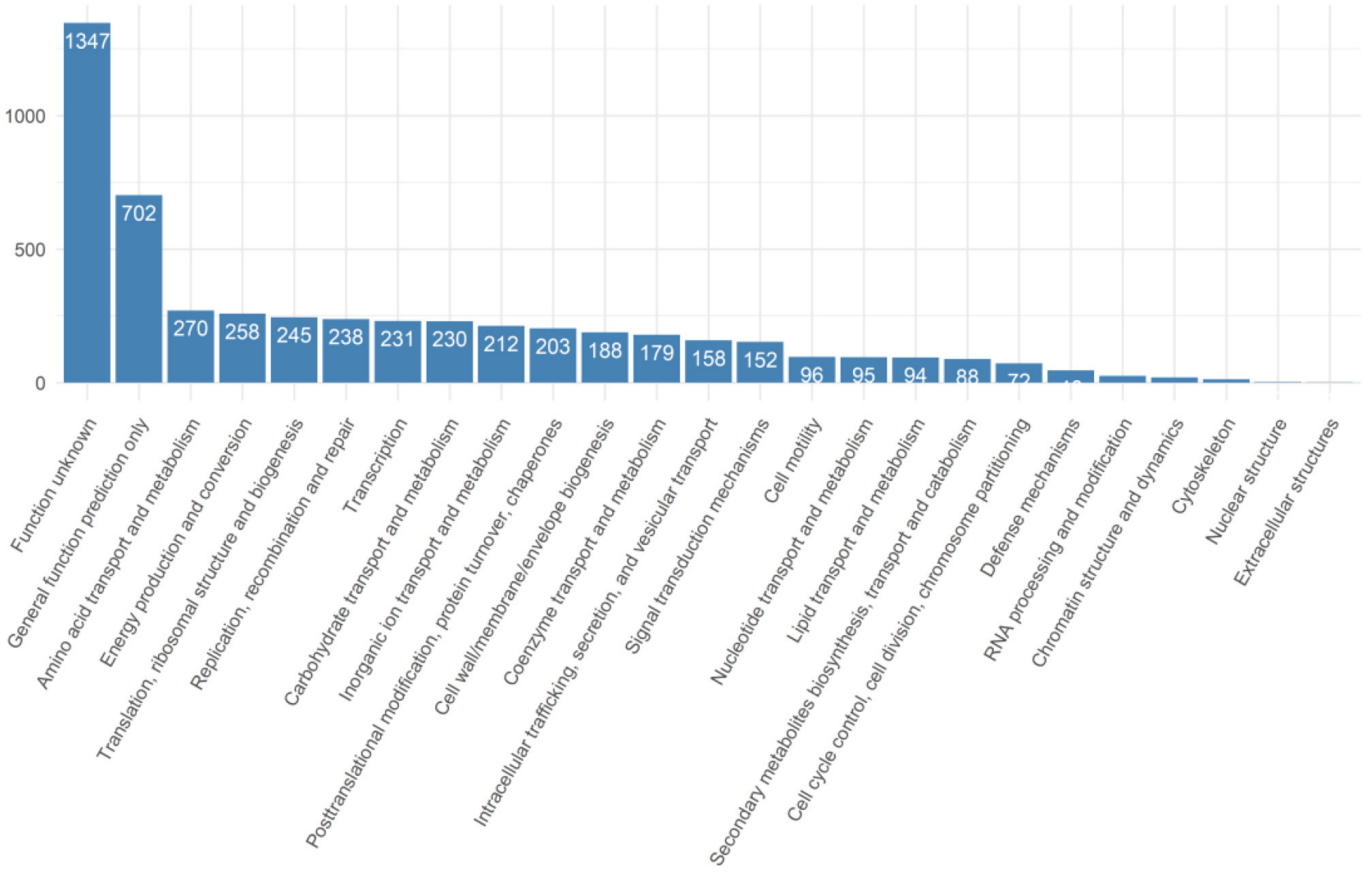
The functional annotation of the genes identified from collected isolates.

A pan-genome of *Salmonella* isolates was constructed by genome comparison among all 40 retail isolates of all three production systems. The genic sections of the isolate genomes were identified to be quite reserved. In total 5,604 genes were identified across the 40 retail *Salmonella* isolates, while 38 isolates share 3,980 core genes, and 1,145 genes were identified shared between six and 38 isolates. In comparison, 479 genes were identified as “cloud genes” that were unique to individual isolates (Figure 1). The functional annotation indicated that the most abundant functional pathways of the bacteria include amino acid transport and metabolism; energy production and conversion; translation and transcription-related activities including ribosomal structure and biogenesis, replication, recombination, and repair; posttranslational modification, protein turnover, and chaperones; and cell wall or membrane-related biogenesis, signaling, and transport. The overview of the functional categories of the gene annotations is given in Figure 2.

A local sequence database of *Salmonella* was constructed, which hosts genome sequences, protein sequences, and annotations. The web-based interface was provided, which enables concurrent enquiries and searches.

### Serotype Drives the Main Genomic Divergence

**Figure 3:**
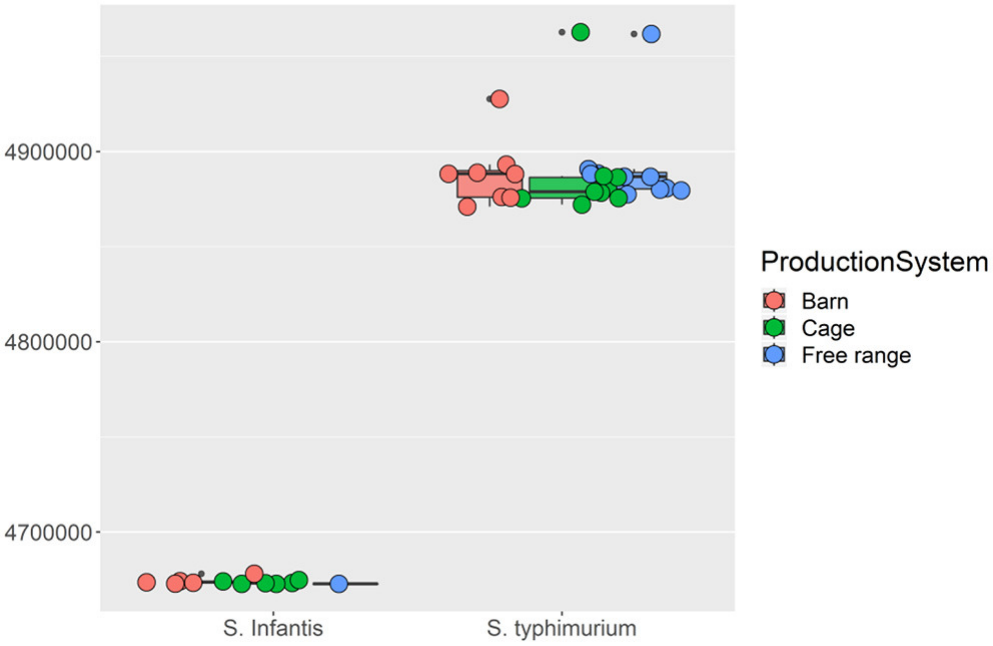
The boxplots of the isolates genome sizes. The *Salmonella* Infantis and *Salmonella* Typhimurium serotypes were grouped separately by the three different egg production systems, namely, barn, cage, and free-range.

**Figure 4:**
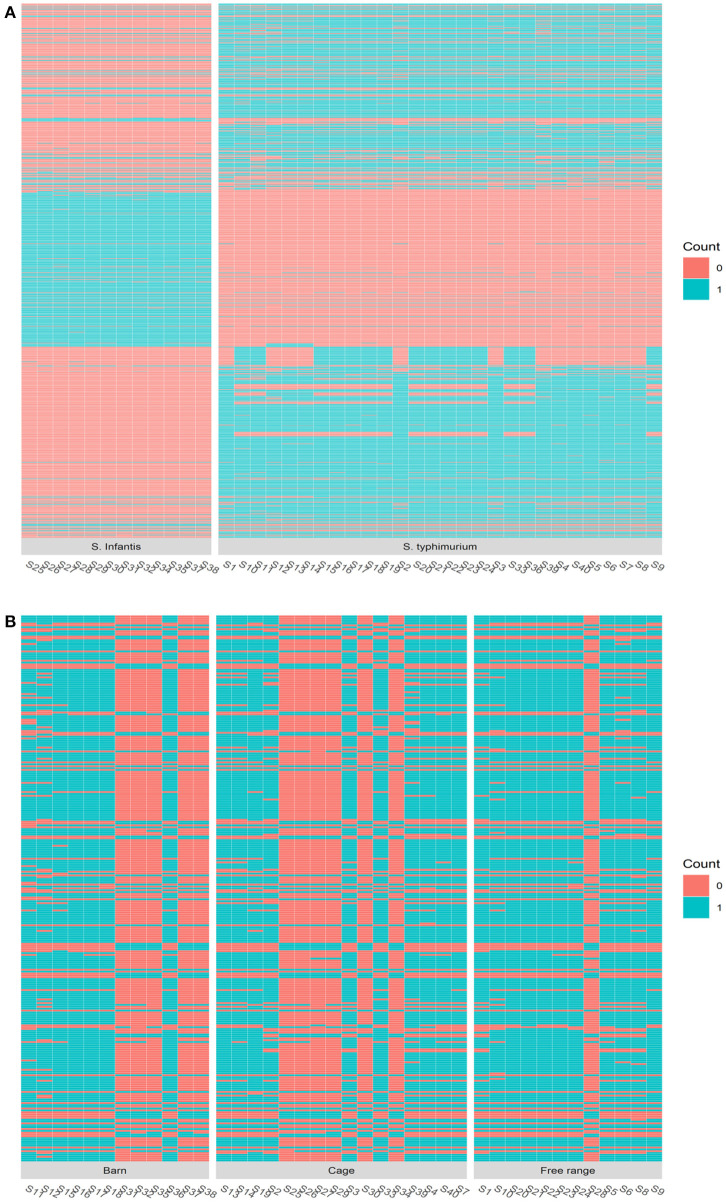
The heatmap of the genomic composition comparison by different factors. **(A)** The heatmap comparing *Salmonella* Infantis and *Salmonella* Typhimurium serotypes on the genes identified to be differentially associated with the serotypic difference. **(B)** The heatmap comparing different egg production systems on the genes identified to be differentially associated with barn, cage, and free-range production systems.

Serotype analysis indicated that the retail isolates consisted of two different serovars: 12 isolates were identified as *Salmonella* Infantis and 28 isolates as *S*. Typhimurium. Interestingly, all of the *S*. Infantis isolates except one were recovered from samples collected from barn and cage production systems, while *S*. Typhimurium isolates were recovered from samples from the free-range production system (*n* = 11) as well as barn and cage (*n* = 17) (Supplementary Table 1). An obvious genetic divergence between these two serotypes can be observed, and this contributes to the majority of the total genomic variations presented by the isolates. Firstly, the genome sizes of *S*. Infantis isolates were consistently smaller than the genome sizes of *S*. Typhimurium isolates (Figure 3). Secondly, the MLST analysis indicates that *S*. Infantis isolates have more MLSTs given their smaller genomes compared with *S*. Typhimurium isolates (Supplementary Table 1). The phylogenetic analysis using maximum likelihood approach on the core gene sets shared by the isolates consistently identified these two serotypes to have different origins. The inferred evolutionary tree is given in Supplementary Figure 2. Based on the gene presence and absence, in total, 924 genes were identified to be differentially associated with the serotypes with Bonferroni-adjusted *p*-values smaller than 0.05. The most consistent genes include *S*. Infantis unique genes (3-oxoacyl-[acyl-carrier-protein] reductase FabG, 50S ribosomal protein L36 2, aldo-keto reductase IolS, and arsenic resistance transcriptional regulator ArsR2, etc.) and *S*. Typhimurium unique genes (2-dehydro-3-deoxy-6-phosphogalactonate aldolase, 2-nitroimidazole transporter, carbohydrate diacid regulator, 3-hexulose-6-phosphate isomerase, 60 kDa SS-A/Ro ribonucleoprotein, abequosyltransferase RfbV, acetylornithine deacetylase, antirestriction protein KlcA, antitoxin CcdA, DinJ, VapB, aspartate aminotransferase, and so on). A heatmap displaying the genetic distribution of the identified differential genes is given in Figure 4A.

### Production System Leads to Genetic Variations

Apart from the serotype difference, the second most important source of the genomic divergence among the isolates was identified to be associated with the egg production systems. Around 40 genes were identified to be able to distinguish the egg production systems by their presence or absence with an adjusted *p*-value < 0.05 and 33 of which have odd ratios >2, which is given in Supplementary Table 2. A heatmap showing the genetic difference presented by the key genes is shown in Figure 4B. The most dominant divergence in gene compositions among the three production systems was observed in the free-range isolates. Our results indicated that the *Salmonella* isolated from eggs sourced from barn and cage production systems were genetically less diverse than the ones sourced from free-range systems. The most distinguishing genes of the isolates recovered from the eggs produced by free-range production system from the isolates recovered from the eggs produced by barn and cage systems include DNA translocase FtsK, l-aspartate oxidase, Macrolide export ATP-binding/permease protein MacB, exodeoxyribonuclease 8, and lysozyme RrrD. These genes were identified to be associated with translocating genes, regulating gene expression, cell wall division and formation, sugar metabolism, and phosphorylation in bacteria (36–42). This demonstrates that the *Salmonella* isolated from eggs source from barn and cage production systems have undergone different mutations that have somehow altered their genomic compositions.

### Production System Associated With Virulence

Searching the assembled genomes against virulence gene database (43), we identified four genes that are associated with virulence, i.e., *SpvB, SpvC, sseL*, and *PhoQ*, belonging to the *Salmonella* plasmid virulence gene family and are mainly involved with the pathogen's toxin and promote the survival and rapid growth of *Salmonella* in the host. Interestingly, these virulence genes were identified in the isolates from barn and cage produced eggs, and free-range isolates were largely free of these virulence genes.

*PhoQ* controls expression of more than 40 genes and is required for intracellular survival, cationic antimicrobial peptides resistance, and stimulation of cytokine secretion. *sseL* gene is a deubiquitinase required for macrophage killing and virulence. The associated genes and their biological pathways are given in Supplementary Figure 3, where the identified virulence genes are marked in red. From Supplementary Figure 3, the detailed regulation mechanisms of the virulence genes can be seen. The *Spv* family genes are involved in the *Salmonella* infection pathway. *SpvB* and *SpvC* are involved in the actin depolymerization by regulating F-actin.

Our analysis has observed little evidence of antimicrobial resistance genes from the *Salmonella* isolates after comprehensive searching through the NCBI's curated Bacterial Antimicrobial Resistance Reference Gene Database, while two isolates were estimated to contain a β-lactamase resistance gene family by ResFinder.

## Discussion

Our *Salmonella* assemblies are of consistent quality across all isolates, capturing well both gene space and repetitive segments across the genome. In total, 1,440 universal orthologs were searched against all chromosomes, with around 94.4% identified by BUSCO (benchmark universal single-copy orthologs) on average (44). This is on par with “Gold Standard” reference genome assemblies, including human (*Homo sapiens* GRCh38) at 95.5%, corn at 97.0% (*Zea mays* W22v2), and (*Arabidopsis thaliana*) at 95.7% (TAIR10). We have constructed a genome sequence database consisting of *S*. Infantis and *S*. Typhimurium isolates collected from retail eggs. Our finding is the first effort in building a comprehensive collection of isolates in WA by collecting samples from retail companies in metropolitan Perth. This study can serve as a valuable genetic resource and roadmap for studying *Salmonella*, tracing infection, and epidemiological surveillance of outbreaks in WA.

Our analysis demonstrated that the egg production system has a reasonable association with the genomic characteristics of the pathogens; different production systems may lead to genetically different pathogens and virulence. We identified around 40 genes that are unique and relevant to the individual production systems; particularly free-range strains appear to be quite genetically divergent from barn and cage strains, which share more similarity in their genomic compositions. The genetic difference of isolates from free-range and the other two systems was significant. Eggs sourced from free-range enterprises were mainly identified to have *S*. Typhimurium, while *S*. Infantis strains were most likely isolated in barn-laid and caged eggs. Our results only identified a handful of virulence genes, but more were found to be present in the *Salmonella* isolates collected from barn and cage production systems compared with the free-range isolates. Previous investigations also identified virulence genes in various *Salmonella* isolates recovered from cage farms (45, 46). However, limited information is available on virulence typing of *Salmonella* serovars isolated from free-range environments (13). There are four virulence genes that were identified almost exclusively from cage and barn eggs. Although the presence of virulence genes may indicate a higher likelihood to cause serious infections in people who contract these strains, the detailed mechanisms of these virulence genes are not fully understood as yet. In recent years, consumer demand for free-range eggs has been fast growing due to the conceived high quality (8). However, limited molecular studies have been performed to scientifically evaluate the safety of free-range eggs. Our results indicated that egg production systems can potentially lead to specific genetic variations in the *Salmonella* isolates. Further research is required to assess how different management practices related to these production systems impact upon the genetic variation (i.e., mutation and virulence) of *Salmonella* serovars.

The difference in genomic profiles and virulence between free-range and the other production systems is likely to be driven by the environmental and management factors. Free-range chicken farms have a lower stocking density than the other two systems with a high stocking density shown to be associated with higher stress, lower immune response, and higher chances of infection (47). These events could provide favorable conditions for bacteria to evolve and develop or sustain virulence factors. In contrast, chickens raised under a free-range production system are more likely to interact with farm's outside environments and intense human contact, as constant interaction is necessary during hand egg collection, cleaning, and monitoring, and herding stock. These events increase the likelihood of uptake of more diverse genes (virulence and resistance). This is very informative and presented a serious argument if certain chicken density requirement be implemented as a regulation for public health policymakers. It would be very interesting to perform more thorough study to evaluate specific environmental factors of different production systems. This will help to identify the most important hazards for fostering bacteria virulence and resistance. Although it is well-known in the plant domain, i.e., cereal crops, canola, and *Arabidopsis*, that the environment plays an equally important role in the genotypes for controlling phenotypes, how the environment affects bacterial genomes is relatively less understood. The results of our study demonstrated that different types of production system for egg laying poultry are associated with the genomic compositions of *Salmonella*.

Our finding indicated very low carriage of antimicrobial resistance genes by all the *Salmonella* strains. No antimicrobial resistance genes have been also identified from *S*. Sofia isolated from chicken meat in a previous study in Australia (48). Finding very few resistance genes in our study provided evidence of the significance of environment in driving bacteria mutations, which might be because of WA is geographically isolated from the rest of Australia and the world or might be due to the strict conservative approach to registration of antibiotics for use in food-producing animals in Australia. As future work, it is recommended to incorporate management strategies and design new ways of quantitatively measure key environmental condition at chicken farms. In this way, the association between the bacteria genetic mutations and the environmental factors can be better understood and modeled. To correlate human *Salmonella* isolates with egg *Salmonella* isolates will be another interesting direction for future research. This kind of integrative analysis may lead to novel methods for predicting the virulence of unseen strains and the resistance to antibiotics so that the treatment and the management can be more effective.

## Data Availability Statement

The datasets generated for this study can be found in online repositories. The names of the repository/repositories and accession number(s) can be found in the article/Supplementary Material.

## Author Contributions

All authors listed have made a substantial, direct and intellectual contribution to the work, and approved it for publication.

## Conflict of Interest

The authors declare that the research was conducted in the absence of any commercial or financial relationships that could be construed as a potential conflict of interest.
