# Supplementary material for: Whole-Genome Comparative Analysis Reveals Association Between Salmonella Genomic Variation and Egg Production Systems
Source: Front Vet Sci. 2021 Jul 12;8:666767. doi: 10.3389/fvets.2021.666767 (PMC8311177; doi:10.3389/fvets.2021.666767)
Supplement: Supplementary file 1 [file Data_Sheet_1.docx]

**Supplementary Table 1.** The detail of *Salmonella* isolates assemblies from retail eggs.

| Name | Total size | N50 | Mean Contig length | Contig no. | Production | Serovar | MLST |
| --- | --- | --- | --- | --- | --- | --- | --- |
| S1 | 4880685 | 225591 | 84149 | 58 | Free-range | *S.* Typhimurium | 19 |
| S2 | 4962671 | 150033 | 22764 | 218 | Cage | *S.* Typhimurium | NA |
| S3 | 4881098 | 270715 | 92096 | 53 | Cage | *S.* Typhimurium | 19 |
| S4 | 4875500 | 130090 | 45995 | 106 | Cage | *S.* Typhimurium | 19 |
| S5 | 4877451 | 99138 | 44340 | 110 | Free-range | *S.* Typhimurium | NA |
| S6 | 4879467 | 169284 | 33421 | 146 | Free-range | *S.* Typhimurium | NA |
| S7 | 4878307 | 208887 | 67754 | 72 | Cage | *S.* Typhimurium | 19 |
| S8 | 4961755 | 155580 | 22865 | 217 | Free-range | *S.* Typhimurium | NA |
| S9 | 4886662 | 382579 | 97733 | 50 | Free-range | *S.* Typhimurium | 19 |
| S10 | 4879943 | 50066 | 25284 | 193 | Free-range | *S.* Typhimurium | 19 |
| S11 | 4876116 | 213825 | 28515 | 171 | Barn | *S.* Typhimurium | NA |
| S12 | 4871033 | 176823 | 34793 | 140 | Barn | *S.* Typhimurium | NA |
| S13 | 4875733 | 270708 | 99504 | 49 | Cage | *S.* Typhimurium | NA |
| S14 | 4875418 | 270708 | 101571 | 48 | Cage | *S.* Typhimurium | 19 |
| S15 | 4886324 | 232117 | 69804 | 70 | Barn | *S.* Typhimurium | 19 |
| S16 | 4888849 | 280020 | 94016 | 52 | Barn | *S.* Typhimurium | NA |
| S17 | 4893099 | 430360 | 80214 | 61 | Barn | *S.* Typhimurium | 19 |
| S18 | 4888195 | 259180 | 82850 | 59 | Barn | *S.* Typhimurium | 19 |
| S19 | 4927648 | 174392 | 42116 | 117 | Cage | *S.* Typhimurium | 19 |
| S20 | 4888413 | 460082 | 104008 | 47 | Free-range | *S.* Typhimurium | 19 |
| S21 | 4886744 | 267138 | 93975 | 52 | Free-range | *S.* Typhimurium | 19 |
| S22 | 4889514 | 266419 | 106293 | 46 | Free-range | *S.* Typhimurium | 19 |
| S23 | 4890778 | 266419 | 99811 | 49 | Free-range | *S.* Typhimurium | 19 |
| S24 | 4888156 | 340540 | 10183 | 48 | Free-range | *S.* Typhimurium | 19 |
| S25 | 4673158 | 277065 | 108678 | 43 | Cage | *S.* Infantis | 32 |
| S26 | 4672733 | 444265 | 186909 | 25 | Cage | *S.* Infantis | 32 |
| S27 | 4672669 | 106885 | 58408 | 80 | Cage | *S.* Infantis | 32 |
| S28 | 4672656 | 279291 | 108666 | 43 | Free-range | *S.* Infantis | 32 |
| S29 | 4673075 | 384981 | 179733 | 26 | Cage | *S.* Infantis | 32 |
| S30 | 4674054 | 444265 | 150775 | 31 | Cage | *S.* Infantis | 32 |
| S31 | 4673599 | 197010 | 119835 | 39 | Barn | *S.* Infantis | 32 |
| S32 | 4678009 | 381441 | 111381 | 42 | Barn | S. Infantis | 32 |
| S33 | 4887057 | 268058 | 82831 | 59 | Cage | S. Typhimurium | 19 |
| S34 | 4674695 | 227659 | 179795 | 26 | Cage | S. Infantis | 32 |
| S35 | 4674173 | 217932 | 194757 | 24 | Barn | S. Infantis | 32 |
| S36 | 4888238 | 258808 | 94004 | 52 | Barn | S. Typhimurium | 19 |
| S37 | 4673359 | 399848 | 186934 | 25 | Barn | *S.* Infantis | 32 |
| S38 | 4672798 | 297245 | 150735 | 31 | Barn | *S.* Infantis | 32 |
| S39 | 4872047 | 130142 | 26335 | 185 | Cage | *S.* Typhimurium | 19 |
| S40 | 4878797 | 225473 | 58780 | 83 | Cage | *S.* Typhimurium | NA |

**Supplementary Table 2.** The list of genes that were identified to be significantly associated with the free-range production system.

| **Gene ID** | **Gene Annotation** | **Odds ratio** | **P-value** |
| --- | --- | --- | --- |
| macB_1 | Macrolide export ATP-binding/permease protein MacB | 13 | 0.00 |
| gene_924 | hypothetical protein | 8.4 | 0.00 |
| gene_911 | hypothetical protein | 12.69231 | 0.01 |
| gene_3467 | hypothetical protein | 9 | 0.01 |
| gene_1132 | hypothetical protein | 11 | 0.01 |
| gene_1461 | hypothetical protein | 11 | 0.01 |
| gene_829 | hypothetical protein | 11 | 0.01 |
| xerD_4 | Tyrosine recombinase XerD | 11 | 0.01 |
| tufA_1 | Elongation factor Tu 1 | 0.090909 | 0.01 |
| gene_1633 | hypothetical protein | 0.090909 | 0.01 |
| gene_1636 | hypothetical protein | 0.090909 | 0.01 |
| gene_1637 | hypothetical protein | 0.090909 | 0.01 |
| gene_1634 | hypothetical protein | 0.090909 | 0.01 |
| gene_1635 | hypothetical protein | 0.090909 | 0.01 |
| gene_1638 | hypothetical protein | 0.090909 | 0.01 |
| gene_1639 | hypothetical protein | 0.090909 | 0.01 |
| traC_1 | DNA primase TraC | 0.090909 | 0.01 |
| gene_406 | L-aspartate oxidase | 0.090909 | 0.01 |
| gene_1725 | hypothetical protein | 0 | 0.01 |
| gene_1071 | hypothetical protein | 9.285714 | 0.01 |
| gene_445 | hypothetical protein | 6.44 | 0.02 |
| gene_595 | hypothetical protein | inf | 0.02 |
| gene_97 | Exodeoxyribonuclease 8 | 13.5 | 0.02 |
| gene_104 | hypothetical protein | 13.5 | 0.02 |
| gene_419 | hypothetical protein | 9.533333 | 0.03 |
| gene_1462 | hypothetical protein | 9.533333 | 0.03 |
| gene_1326 | hypothetical protein | 9.533333 | 0.03 |
| gene_1460 | hypothetical protein | 9.533333 | 0.03 |
| dsbA_2 | Thiol:disulfide interchange protein DsbA | 9.533333 | 0.03 |
| traJ | Protein TraJ | 9.533333 | 0.03 |
| traQ | Protein TraQ | 9.533333 | 0.03 |
| traR | Protein TraR | 9.533333 | 0.03 |
| gene_185 | hypothetical protein | 9.533333 | 0.03 |
| gene_1170 | hypothetical protein | 9.533333 | 0.03 |
| pdeL | Cyclic di-GMP phosphodiesterase PdeL | 9.533333 | 0.03 |
| gene_442 | hypothetical protein | 9.533333 | 0.03 |
| gene_426 | hypothetical protein | 9.533333 | 0.03 |
| gadX_3 | HTH-type transcriptional regulator GadX | 9.533333 | 0.03 |
| gene_60 | DNA translocase FtsK | 0.15 | 0.03 |
| lpfD_1 | putative minor fimbrial subunit LpfD | inf | 0.03 |
| gene_1853 | hypothetical protein | 0 | 0.03 |
| gene_408 | hypothetical protein | 5.952381 | 0.038574 |
| smc_3 | hypothetical protein | 5.769231 | 0.040845 |
| gene_1073 | hypothetical protein | 5.769231 | 0.040845 |
| recE_2 | hypothetical protein | 5.769231 | 0.040845 |
| gene_1 | O-acetyl-ADP-ribose deacetylase | 0.173333 | 0.040845 |


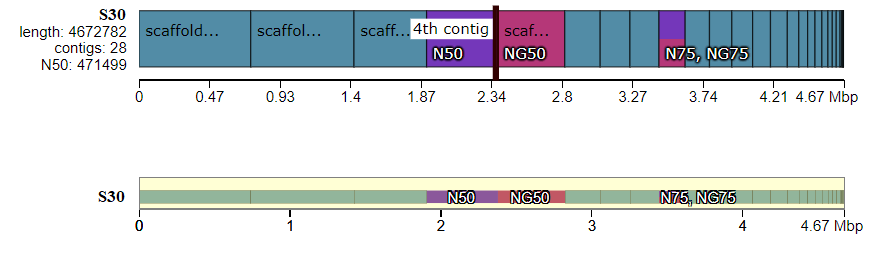


**Supplementary Fig 1.** The assembly statistics of sample S30 a retailed isolate from cage production system. Less than 4 contigs have reached more than 50% of the assembly.

**A.**


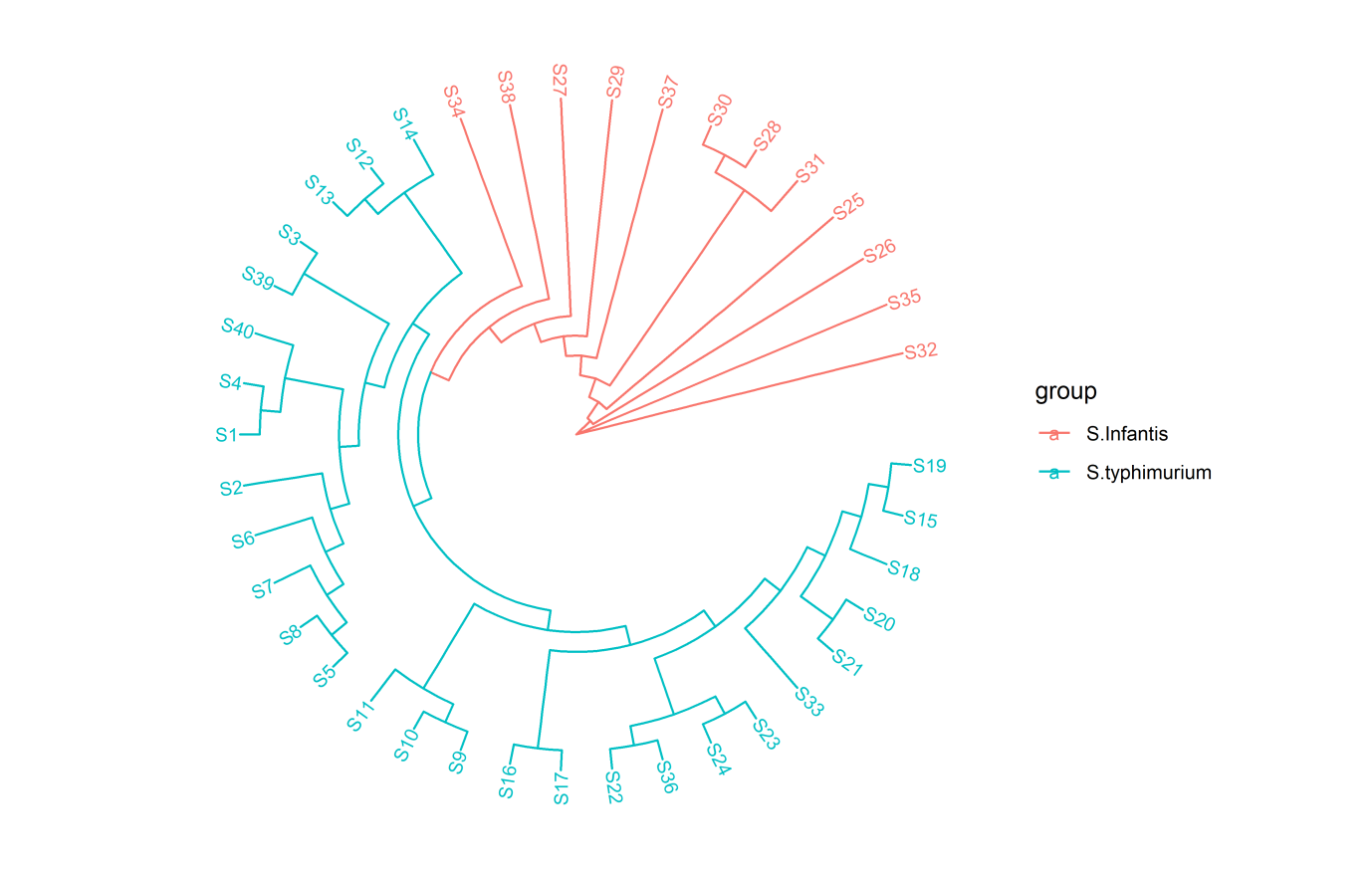


**B.**


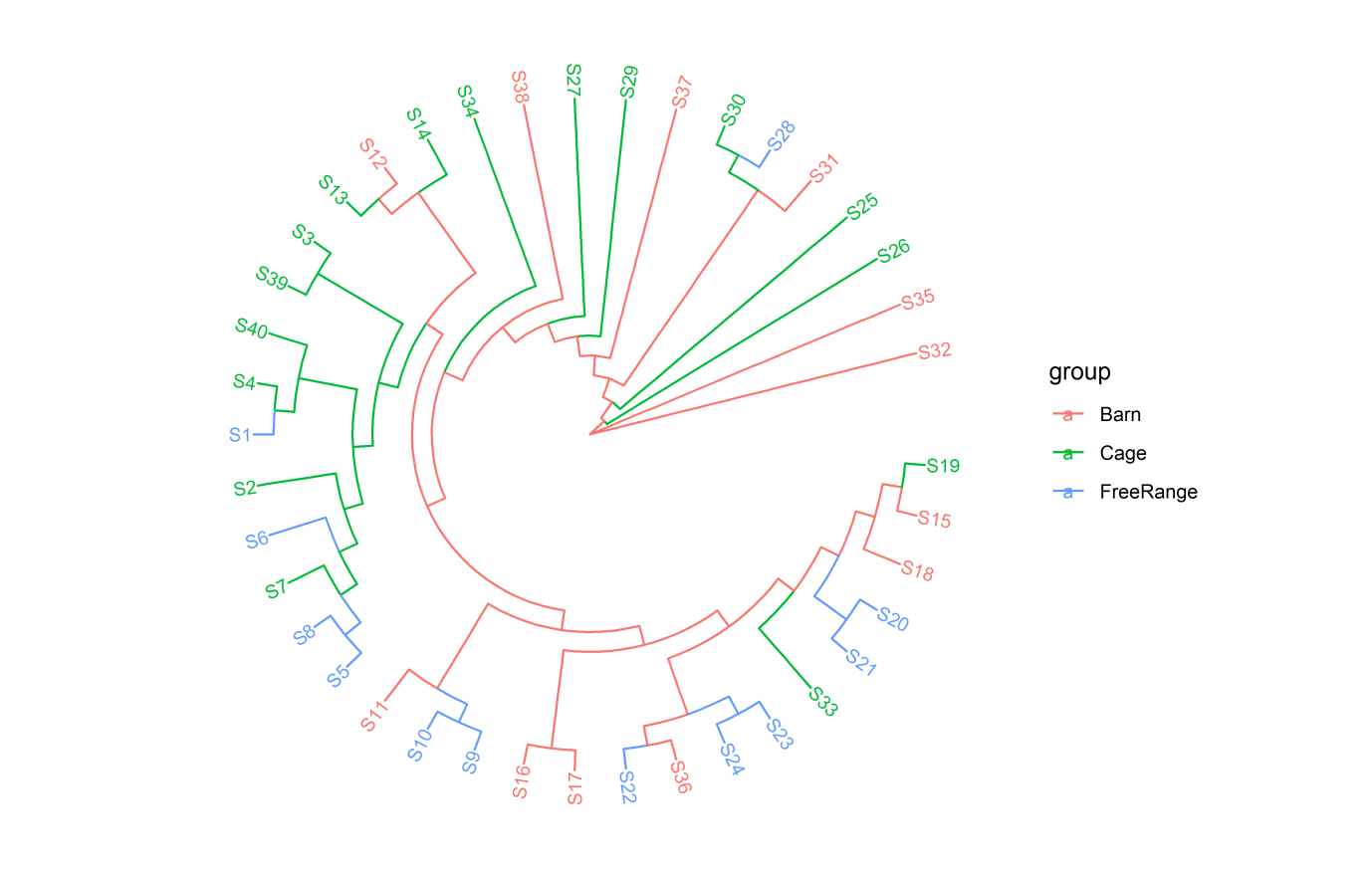


**Supplementary Fig 2.** The inferred phylogenetic tree by maximum likelihood approach on the core 3923 genes that were shared by the isolates. **A.** the isolates were coloured by their serotype; **B.** the isolates were marked by the production systems.

**A.**


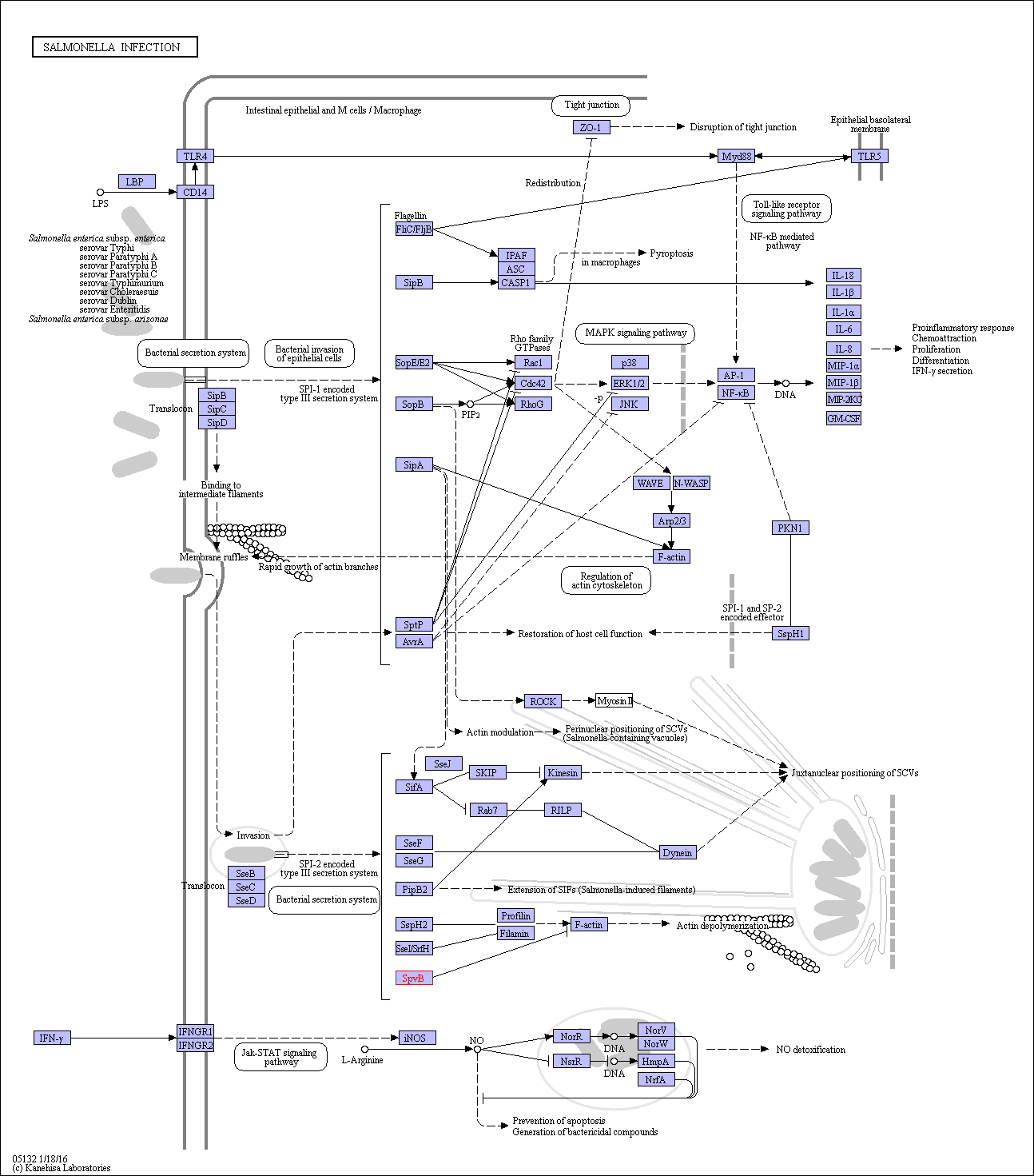


**B.**


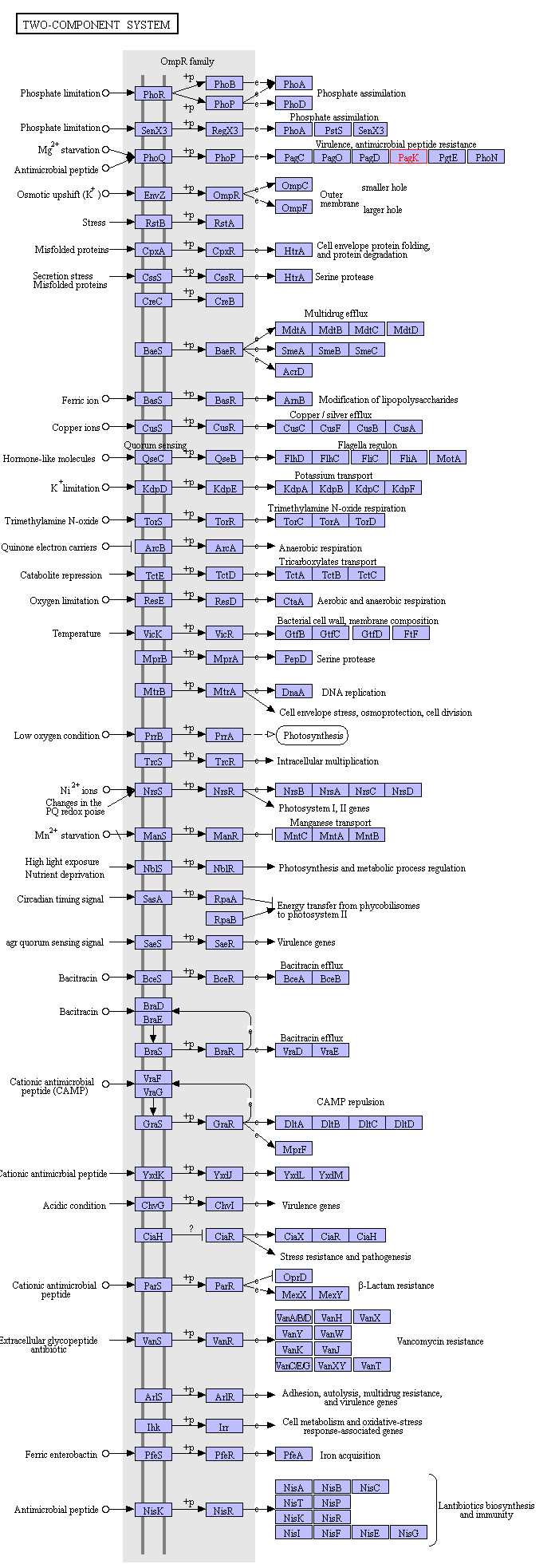


**Supplementary Fig 3.** The pathways diagrams associated with the key identified virulence genes. The detailed regulating mechanisms of the virulence genes can be seen. Identified virulence genes were marked in red. **A.** The pathway of *Salmonella* infection where the identified *SpV* family virulence genes (*SpvB* and *SpvC*) is associated with the Actin depolymerization by regulating F-actin. **B.** demonstrates the two-component signal transduction systems and how the identified virulence gene *PhoQ* is an up-stream regulating gene for the whole system.
